# Complexity Science in Human Change: Research, Models, Clinical Applications

**DOI:** 10.3390/e24111670

**Published:** 2022-11-17

**Authors:** Franco Orsucci, Wolfgang Tschacher

**Affiliations:** 1 Department of Psychology, University College London, London WC1E 6BT, UK; 2 Norfolk and Suffolk NHS Foundation Trust, Drayton High Road, Norwich NR6 5BE, UK; 3 Department of Experimental Psychology, University Hospital of Psychiatry and Psychotherapy, CH-3060 Bern, Switzerland

Complexity and entropy prevail in human behavior and social interaction because the systems underlying behavior and interaction are, without a doubt, highly complex. The human brain, body, language, society, and culture consist of vast numbers of components, and the degrees of freedom in behavior, cognition, and experience are just as immense. So why do we usually experience the world around us as structured and well-organized instead of disorganized and random? How do the patterns emerge? We are witnessing self-organization and pattern-formation processes, which organize and modulate complexity.

Increasingly, such processes are acknowledged as essential for human affairs and are gradually coming to the fore in psychology and social science research. Research informed by dynamical systems theory, synergetics, and complexity theory has introduced concepts such as attractor, synchrony, and coupling to psychology. In psychotherapy research, empirical findings show that regular patterns of interaction arise in all therapeutic relationships. The therapist–patient alliance is a paradigmatic case to highlight further how interactions evolve and can be changed and how humans can change. Attractors describe the stable states of a process, e.g., the stability or instability of personalities and disorders. They can be detected and described based on empirical time-series.

In the first paper, Orsucci [1] examines certain theoretical implications of empirical studies developed over recent years by his research groups. These experiments have explored the biosemiotic nature of communication streams from emotional neuroscience and embodied mind perspectives. Information combinatorics analysis enabled a deeper understanding of the coupling and decoupling dynamics of biosemiotics streams. They investigated intraindividual and interpersonal relations as the coevolution dynamics of hybrid couplings, synchronizations, and desynchronizations. Cluster analysis and Markov chains produced evidence of chimera states and phase transitions. A probabilistic and nondeterministic approach clarified the properties of these hybrid dynamics. As a result, multidimensional theoretical models can better represent the hybrid nature of human interactions.

In the second contribution, a study by Tomashin et al. [2], the authors consider how fractal properties in time series of human behavior and physiology are ubiquitous, and several methods to capture such properties have been proposed in the past decades. The paper takes this suggestion as a point of departure to propose and test several approaches to quantifying fractal fluctuations in synthetic and empirical time-series data using recurrence-based analysis. They show that such measures can be extracted based on recurrence plots and contrast the different approaches in terms of their accuracy and range of applicability.

In the third paper, Altmann et al. [3] compare eight algorithms that quantify synchronization in time series. The authors use a benchmark dataset that describes body movement in 30 dyadic interviews on somatic complaints conducted by medical students with 15 depressed and 15 healthy interviewees. Twenty-one different synchrony measures are tested, derived from four classes of algorithms: (windowed) cross-correlations, local regressions with or without peak-picking, mutual information, and cross-recurrence quantification. The intercorrelations of the results show that synchrony estimations are highly divergent, and no convergent validity is manifest. However, measures from the same class tend to be correlated, and cross-correlation-based measures form a factor. In contrast, the mutual information and the peak-picking measures load on a different factor. Most measures do not support the assumption underlying predictive validity that depression should have lowered synchrony measures. The authors conclude that more analyses and sensitivity studies are needed to clarify the psychological meaning of synchrony.

In the fourth contribution, research by Stamovlasis et al. [4], the authors investigate and propose a nonlinear model that might explain empirical data better than ordinary linear ones and elucidate the role of depression in a financial capacity. Financial incapacity is one of the cognitive deficits observed in amnestic mild cognitive impairment and dementia, while the combined interference of depression remains unexplored. Cusp catastrophe analysis was applied to the data, which suggested that the nonlinear model was superior to the linear and logistic alternatives, demonstrating that depression contributes to a bifurcation effect. Depressive symptomatology induces nonlinear effects, and a sudden decline in financial capacity is observed beyond a threshold value. Implications for theory and practice are discussed.

In the fifth contribution, Zubek et al. [5] reflected on the pandemic that forced our daily interactions to move into the virtual world. People had to adapt to new commu-nication media that afford different ways of interaction. Remote communication de-creases the availability and salience of some cues but also may enable and highlight others. Importantly, basic movement dynamics, which are crucial for any interaction as they are responsible for the informational and affective coupling, are affected. It is therefore essential to discover exactly how these dynamics change. In this exploratory study of six interacting dyads, they used traditional variability measures and cross re-currence quantification analysis to compare the movement coordination dynamics in quasi-natural dialogues in four situations: (1) remote video-mediated conversations with a self-view mirror image present, (2) remote video-mediated conversations without a self-view, (3) face-to-face conversations with a self-view, and (4) face-to-face conversations without a self-view. They discovered that in remote interactions move-ments pertaining to communicative gestures were exaggerated, while the stability of interpersonal coordination was greatly decreased. The presence of the self-view image made the gestures less exaggerated but did not affect the coordination. The dynamical analyses clarified the interaction processes and may be useful in explaining phenomena connected with video-mediated communication, such as “Zoom fatigue”.

In the sixth contribution, Laudańska et al. [6] clarify how infants’ limb movements evolve from disorganized to more selectively coordinated during the first year of life as they learn to navigate and interact with an ever-changing environment more efficiently. However, how these coordination patterns change during the first year of life and across different contexts is unknown. Here, they used wearable motion trackers to study the developmental changes in the complexity of limb movements (arms and legs) at 4, 6, 9, and 12 months of age in two different tasks: rhythmic rattle-shaking and free play. They applied multidimensional recurrence quantification analysis (MdRQA) to capture the nonlinear changes in infants’ limb complexity. They show that the MdRQA parameters (entropy, recurrence rate, and mean line) are task-dependent only at 9 and 12 months of age, with higher values in rattle-shaking than free play. Infants’ motor system becomes more stable and flexible with age, allowing for the flexible adaptation of behaviors to task demands.

The seventh contribution by Ganesh and Gabora [7] take a human dynamical systems approach to modeling therapeutic change, using reflexively autocatalytic food set-derived (RAF) networks. RAFs have been used to model the self-organization of adaptive networks associated with the origin and early evolution of both biological life and the development of the kind of cognitive structure necessary for cultural evolution. The RAF approach is applicable in these seemingly disparate cases because it provides a theoretical framework for formally describing under what conditions systems composed of elements that interact and “catalyze” the formation of new elements collectively become integrated wholes. This contribution develops in line with the growing recognition of the role of embodiment, affect, and implicit processes in psychotherapy, and several recent studies have examined the role of physiological synchrony in the process and outcome of psychotherapy. This study aims to introduce partial directed coherence (PDC) as a novel approach to calculating psychophysiological synchrony and examines its potential to contribute to our understanding of the therapy process. The study adopts a single-case, mixed-method design and examines physiological synchrony in one-couple therapy in relation to the therapeutic alliance and a narrative analysis of meaning construction in the sessions. The findings of this study point to the complex interplay between explicit and implicit levels of interaction and the potential contribution of including physiological synchrony in the study of interactional processes in psychotherapy.

The paper by Avdi et al. [8] addresses physiological synchrony in one family therapy of fifteen sessions and two physiological measurement sessions in which cardiac measures were recorded. The sessions concern a couple and two female psychotherapists. Physiological data are transformed into an index of sympathetic activity, and synchrony is computed using partial directed coherence. This method detects the direction of influence (“pacing/leading”) in each pair of participants. In addition to the quantitative findings on synchrony, rating scales depict therapeutic alliance, and qualitative coding separates the measurement sessions into topical episodes that are semantically similar. Finally, the therapy process is described by the percentage of time windows synchronized concerning the couple’s sympathetic activity, which is found to be reduced in the second measurement session, where the patterns of pacing and leading have changed towards a more balanced embodied relatedness. The authors conclude that a mixed-methods approach allows the linking of the quantitative synchrony findings to the qualitative clinical process in this successful couple therapy.

In the ninth paper, a study by Nkomidio et al. [9], the authors investigate the response characteristics of a two-dimensional neuron model exposed to an externally applied extremely low frequency (ELF) sinusoidal electric field and the synchronization of neurons weakly coupled with gap junction. They find, by numerical simulations, that neurons can exhibit different spiking patterns, which are well observed in the structure of the recurrence plot (R.P.). Then they further study the synchronization between weakly coupled neurons in chaotic regimes under the influence of a weak ELF electric field. In general, detecting the phases of chaotic spiky signals is not easy when using standard methods. Recurrence analysis provides a reliable tool for defining phases, even for noncoherent regimes or spiky signals. Recurrence-based synchronization analysis reveals that, even in the range of weak coupling, the phase synchronization of the coupled neurons occurs. By adding an ELF electric field, this synchronization increases depending on the amplitude of the externally applied ELF electric field. Authors further suggest a novel measure for RP-based phase synchronization analysis, which better considers the probabilities of recurrences.

In the tenth contribution, Webber [10] clarifies how the recurrence analyses of dynamical systems can only process the short sections of signals that may be infinitely long. By necessity, the recurrence plot and its quantifications are constrained within a truncated triangle that clips the signals at its borders. Recurrence variables defined within these confining borders can be influenced by truncation effects depending on the system under evaluation. In this study, the question being asked is, if the boundary borders were tilted, what would be the effect on all recurrence variables? This question is examined by comparing recurrence variables computed with the triangular recurrence area versus the boxed recurrence area. Examples include the logistic equation (mathematical series), the Dow Jones Industrial Average over a decade (real-world data), and a square wave pulse (toy series). Good agreement among the variables in terms of timing and amplitude was found for most, but not all, variables. These significant results are discussed.

In the eleventh paper, Ciompi and Tschacher [11] develop an account of modeling schizophrenia based on four different but related complexity theories: affect-logic, 4E-cognition/embodiment, synergetics, and the free-energy principle. All theories have in common that they are built on loop dynamics, so-called circular causality. In affect-logic, the loop is given by circular interactions between emotion (‘affect’) and cognition (‘logic’), where emotion is the energy source for cognitive dynamics. Such interactions occur at the individual level, the level of micro-social interaction, and the societal level, which are structurally coupled. In synergetics, emotions act as control parameters that drive the system toward pattern formation. The embodiment and the free-energy principles are likewise built on circular dynamics between mind and body, respectively, between a generative model and sensory evidence. The article uses these commonalities for insights into the dynamics of schizophrenia spectrum symptoms: overly strong emotional tension then forces the cognitive system into dysfunctional patterns, which, however, are functional insofar as free energy is reduced. Ideas for therapeutic guidelines, as in the Soteria model, are also derived.

In the twelfth paper, Prinz et al. [12] compute the synchrony of electrodermal activity in psychotherapy interventions, focusing on the technique of imagery rescripting. This therapeutic technique was developed to modulate traumatic memories in a positive and desired direction. The activation of such memories commonly also affects the therapist involved in the session. Therefore, client-therapist synchrony based on cross-correlations is explored in 50 clients. Client-led synchrony is differentiated from therapist-led synchrony by the sign of the lags of cross-correlations. It is found that therapist-led synchrony is significantly associated with clients’ emotional experiences of greater contentment, lower anxiety, and lower depression. In contrast, client-led synchrony is linked to the clients’ more significant anxiety. The authors interpret their findings as supportive of the therapists’ role in regulating mood.

In their contribution, Gennaro et al. [13] introduce a novel lexical method called the affective saturation index (ASI) to assess affectivity based on interview transcripts. Affect is semiotically defined as a sign that makes sense of the world in terms of patterns of bodily activation. Affect saturation in the ASI is then defined based on a “phase space of meaning”. The ASI was correlated with several measures of semantic complexity, students’ emotion regulation, and heart-rate variability in a sample of 40 students who participated in semi-structured interviews on neutral issues. The study shows that affective saturation is significantly and inversely linked to the semantic entropy index and heart-rate variability, consistent with the expectation that the ASI can detect the lexical-syntactic complexity of the interview text as well as physiological signs of affective arousal. It is concluded that ASI thus has potential applicability in clinical and community interventions, social communication, marketing, and media monitoring. Most approaches to computing interpersonal coupling are dyadic in that they focus on bivariate synchrony, such as that between client and therapist.

Meier and Tschacher [14] developed an algorithm for multivariate surrogate synchrony (mv-SUSY), which is based on the eigen-decomposition of the correlation matrix of multiple time series, like principal component analysis (mv-SUSY variant lambdamax). A further variant labeled omega is derived from the determinant of the variance-covariance matrix as a measure of actual entropy and standardized by potential entropy, the product of all variances. Computation is carried out in time-series segments, and segment-shuffled surrogate datasets are used as a control condition. The authors apply mv-SUSY to the simulated multivariate time series that realize the various types of regularities (random data, autocorrelations, trends, oscillatory behavior, intercorrelated random data) and to empirical multivariate time series (motion capture data from persons dancing and from a group discussion). It was found that mv-SUSY correctly identifies whether regular patterns exist in the datasets. The results of the multivariate algorithms are additionally validated by conventional dyadic synchrony methods.

We believe that change is generally studied in phase transitions when the dynamics move between different attractors, as evident in behaviors, mental states, and neurobiology. Theoretical models can represent dynamical change maps in mathematical equations and topological structures. Mapping theory to empirical research and vice versa is challenging but heuristic. Nevertheless, it paves the road to a future discipline of a general complexity theory of human change.

One feature of complexity and self-organization is the presence of scaling and fractal dynamics with the emergence of higher-order organizations. Moreover, heterogeneous human networks present specific kinds of self-similarity in the embodied mind in individual and social dynamics. Finally, translational processes and procedures from research to applications and vice versa are particularly relevant as they frequently include interdisciplinary collaborations.

Based on these thoughts, the Special Issue “Complexity Science in Human Change” has addressed an interdisciplinary community of scientists and practitioners interested in dynamical systems theory, especially approaches considering complex systems and applications to psychology and psychotherapy. Most of the contributions in this Issue analyze empirical data, predominantly time series. Some contributions contain theoretical models or methodological topics of complexity science.

This Special Issue closely represents the current work of complexity researchers in human behavior and change. Psychotherapy and communication systems are the backgrounds of six articles, mental health and psychopathology of four articles, and one paper concerns developmental psychology. Six papers put forward methods for the computation of interpersonal synchrony and innovations of existing synchrony algorithms. Regarding methodology, recurrence quantification analysis is frequently applied in articles on this topic. Four put forward correlation-based analyses of synchrony. Finally, network modeling, catastrophe theory, and nonlinear regression are tackled in one article.

This Special Issue highlights achievements of complexity science in studying patterns of organization and change in human dynamics. It also highlights new challenges that lie ahead. First, it clarifies how complex systems present plural structural forms and varieties of organization and disorganization [15,16]. These varieties are frequently distributed even within any singular system, creating rugged dynamical landscapes. This applies more specifically to human systems, which are hybrid by default [17]. They present multiple scales and heterogeneous subsystems; synchronous and asynchronous interactions; stable, unstable, and metastable states; and localized and generalized dynamics. Therefore, considering the hyper-complexity of the human dynamical landscapes, empirical studies can use mixed methods with several different approaches (sometimes all at once). Accordingly, multiple and varied interventions can induce change or facilitate its natural evolution and emersion [18,19]. This can explain how multiple and different therapeutic techniques can produce similar (though not identical) outcomes in the clinical field. It is the good old equifinality principle of complex open systems still at work.

We hope that with this, new questions and new research might be incited, as Voltaire once suggested: “The most useful books are those in which the readers themselves supply half of the meaning”.
